# Workplace risk factors for anxiety and depression in male-dominated industries: a systematic review

**DOI:** 10.1080/21642850.2014.954579

**Published:** 2014-10-06

**Authors:** Samantha Battams, Ann M. Roche, Jane A. Fischer, Nicole K. Lee, Jacqui Cameron, Victoria Kostadinov

**Affiliations:** ^a^ National Centre for Education and Training on Addiction, Flinders University, Adelaide, GPO Box 2100 Adelaide, SA5001, Australia

**Keywords:** anxiety, depression, male-dominated industry, risk factors, workplace

## Abstract

*Background and Aims*: Working conditions are an important health determinant. Employment factors can negatively affect mental health (MH), but there is little research on MH risk factors in male-dominated industries (MDI). *Method*: A systematic review of risk factors for anxiety and depression disorders in MDI was undertaken. MDI comprised ≥ 70% male workers and included agriculture, construction, mining, manufacturing, transport and utilities. Major electronic databases (CINAHL, Cochrane Library, Informit, PsycINFO, PubMed and Scopus) were searched. Each study was categorised according to National Health and Medical Research Council's hierarchy of evidence and study quality was assessed according to six methodological criteria. *Results*: Nineteen studies met the inclusion criteria. Four categories of risk were identified: individual factors, team environment, work conditions and work–home interference. The main risk factors associated with anxiety and depression in MDI were poor health and lifestyles, unsupportive workplace relationships, job overload and job demands. Some studies indicated a higher risk of anxiety and depression for blue-collar workers. *Conclusion*: Substantial gaps exist in the evidence. Studies with stronger methodologies are required. Available evidence suggests that comprehensive primary, secondary and tertiary prevention approaches to address MH risk factors in MDI are necessary. There is a need for organisationally focused workplace MH policies and interventions.

## 1. Introduction

The nature of a person's work, and the context and setting in which that work is performed, can have a substantial impact on their mental health (MH) (Faragher, Cass, & Cooper, 2005; Maslach, 2003; Segerstrom & Miller, 2004; Stansfeld & Candy, 2006). Work-related factors, including job demands and social support in the workplace, are particularly important for MH (Butterworth et al., 2011; Kuoppala, Lamminpää, & Husman, 2008; Meltzer et al., 2010). Workplace physical injuries have also been shown to increase the risk of MH problems (Asfaw & Souza, 2012). Poor work conditions have been associated with poorer MH among workers compared with those who are unemployed (Butterworth et al., 2011).

There are numerous social and economic imperatives to reduce the prevalence of MH problems within the working population. Costs borne by the workplace as a result of suboptimal employee MH, including those due to absenteeism and loss of worker productivity, can be substantial (Conti & Burton, 1994; Dewa & Lin, 2000). In the USA, workers with depression have been estimated to cost employers $44 billion per year in lost productivity (Stewart, Ricci, Chee, Hahn, & Morganstein, 2003). Poor MH is also estimated to be associated with 50–60% of all workplace absenteeism (Milczarek, Schneider, & Rial González, 2009).

MH problems contribute significantly to the global burden of disease. They are the largest contributor to years lost due to disability, particularly depression and anxiety, which contribute 2.5% and 1.1%, respectively (Murray et al., 2012). From 1990 to 2010, major depressive disorders increased from the fifteenth to eleventh ranked position (a 37% increase) as a cause of Disability Adjusted Life Years (Murray et al., 2012).

Depression and anxiety are also the most common MH problems. Globally, anxiety disorders (12-month prevalence 2.4–18.2%) and mood disorders (12-month prevalence 0.8–9.6%) are most common (Demyttenaere et al., 2004), with pooled 1-year prevalence rates of 10.6% and 4.1%, respectively (Somers, Goldner, Waraich, & Hsu, 2006; Waraich, Goldner, Somers, & Hsu, 2004).

In the USA, 7% of full-time workers experienced a major depressive episode in the past year (Substance Abuse and Mental Health Services Administration, 2007). The prevalence of MH disorders among workers in developed countries ranges from 11–19% in Australia (Hilton & Whiteford, 2010), and 13% in the UK (Stansfeld, Rasul, Head, & Singleton, 2011), to 3% for anxiety and 13% for depression in Canada (Thompson, Jacobs, & Dewa, 2011). In the USA, anxiety, stress and neurotic disorders were responsible for the greatest number of days off work (National Institute for Occupational Health and Safety, 2004) and in the UK, occupational stress was the second highest cause of absenteeism for non-manual workers (Giga, Noblet, Faragher, & Cooper, 2003). In a recent American study, 13% of the population, including 1 in 4 women aged 50–64 years, was on anti-depressants (Zhong et al., 2013), and an Australian study of 92,000 workers found that 65% of clinically depressed employees did not seek treatment (Whiteford, Sheridan, Cleary, & Hilton, 2005). Similarly, the prevalence rate for anti-depressant use in Australia doubled during 2000–2011 (Stephenson, Karanges, & McGregor, 2013).

While in the general population women have higher rates of anxiety and depression than men (F: 22% vs. M: 18%), workers in a number of MDI have higher than average rates of anxiety and mood disorders. MDI are those where more than 70% of workers are men (Australian Bureau of Statistics, 2008a); in Australia, these industries include agriculture, construction, mining and utilities, which have mental disorder prevalence rates of 20.6%, 23.3%, 22.4%, 20.7%, respectively (Australian Bureau of Statistics, 2008b). Men are often reluctant to seek help or delay seeking help for health problems, especially MH problems (Addis & Mahalik, 2003; Barney, Griffiths, Jorm, & Christensen, 2006; Galdas, Cheater, & Marshall, 2005), which may explain their higher than average prevalence in these industries.

In a meta-analytic review, Stansfeld and Candy (2006) found that a combination of both high demands and low decision latitude in the workplace with high demands and low rewards, were risk factors for MH problems. The high prevalence of anxiety and depression among workers in MDI (Australian Bureau of Statistics, 2008a) suggests that these factors may be especially salient within such industries, or that there are specific factors that increase the risk of MH issues among workers in these industries.

The objective of this systematic review was to examine the risk factors for anxiety and depression among workers in MDI. A broad approach was utilised because, to date, no synthesis of studies related to these risk factors in MDI has been undertaken.

## 2. Methods

### 2.1. Definition of a male-dominated industry

A male-dominated industry has been defined by the Australian and New Zealand Standard Industrial Classification (Australian Bureau of Statistics, 2008a) as one in which there are predominantly male workers, that is, ≥ 70% male workers. In Australia, these industries are agriculture, construction, mining, manufacturing, transport and utilities (Table 1).

**Table 1.** Australian industries comprised of high proportions of male workers.

Industries	Total workforce (N)	Male (%)
Agriculture	249,828	70.0
Construction	828,912	87.8
Mining	176,562	82.6
Manufacturing	902,830	74.0
Transport	479,181	76.8
Utilities	115,610	76.1

Source: Australian Bureau of Statistics (2006).

### 2.2 Eligibility criteria

Studies examining risk factors for anxiety and depression disorders in MDI, published between January 1990 and June 2012 in English, with adult male and/or female participants in paid work were included in the review. Studies were included if they contained measures of depression or anxiety, or where participants were diagnosed with anxiety and/or depression by clinicians (e.g. based on health insurance claims). Studies were excluded if they primarily investigated MH issues other than anxiety and depression, or did not include workers in one of the six identified MDI.

The studies reviewed included a range of clinical diagnostic scales (e.g. the DSM-111-R and DSM-IV-R) and self-rated depression and anxiety scales (e.g. Zung Self-rated Depression Scale, items on the General Health Questionnaire (GHQ)). Clinical depression and anxiety rating scales are generally designed to detect the presence of disorders that meet criteria for a diagnosis and are generally modelled on the criteria in one of the recognised diagnostic systems. While rating scales do not provide a diagnosis per se, they are generally validated against diagnostic instruments, such as the Composite International Diagnostic Interview (CIDI) or expert clinical interview, and correspond well with the diagnosis of depression or anxiety. As they are highly correlated with a diagnosis, we have used the general terms anxiety/depression. This is intended to connote ‘clinically significant’ anxiety/depression to distinguish it from less severe, transient symptoms of anxiety/depression that are not likely to require intervention.

### 2.3. Search strategy

Searches were conducted using the electronic databases: CINAHL, Cochrane Library, Informit, PsycINFO, PubMed and Scopus. Searches combined MeSH and other database thesaurus headings, Boolean terms and keywords. Hand searches of study reference lists and searches of the grey literature were also conducted using conventional electronic search engines, such as Google.

### 2.4. Study selection

**Figure 1.**
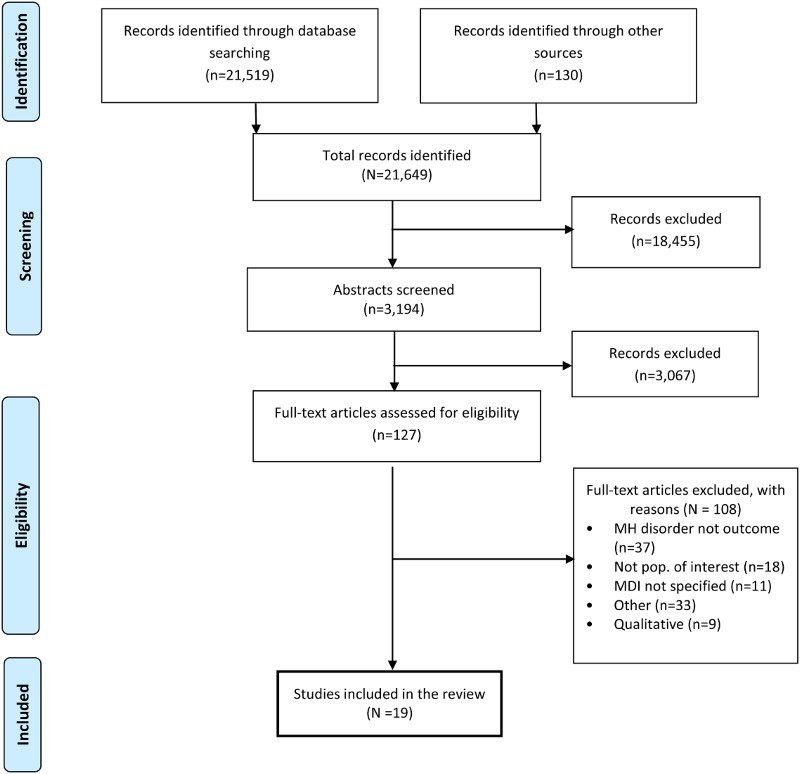
Flow diagram of study selection for systematic review of published research on MH risk factors in MDI.

Studies identified in the initial search underwent a two-stage screening process to ensure that they met the inclusion criteria. First, two reviewers screened each article title and abstract for relevance. At the second screen, one reviewer checked the full article. Excluded papers were screened by a senior reviewer. Figure 1 displays the studies remaining at each step.

### 2.5. Quality assessment

Study quality was assessed in two ways. Each study's level of evidence was considered against the National Health and Medical Research Council's (NHMRC) evidence hierarchy (1999, 2000). The NHMRC evidence hierarchy consists of six levels: I (systematic reviews), II (prospective cohort studies), III-1 (representative samples), III-2 (retrospective cohort studies), III-3 (case–control studies) and IV (a cross-sectional or case series studies).

Studies were also assessed as being either ‘strong, moderate or weak’ after a thorough analysis based on the *Quality Assessment Tool for Quantitative Studies – Effective Public Health Practice Project* (National Collaborating Centre for Methods and Tools, 2009). This tool includes consideration of: (1) selection bias; (2) study design; (3) confounders; (4) blinding (for RCTs); (5) data collection methods; (6) withdrawals and dropouts; (7) intervention integrity (where appropriate); and (8) analyses. Given the nature of studies reviewed (there were no experimental studies), blinding and intervention integrity were not considered. Based on scores for the remaining six factors, a global rating for each study was developed using the quality assessment tool.

Studies that obtained at least four ratings of strong, with no ratings of weak for any of the assessment criteria, were assessed as methodologically strong. Studies that obtained less than four strong ratings but no more than one weak rating for any of the assessment criteria were assessed as methodologically moderate. Studies that obtained two or more weak ratings for any of the assessment criteria were assessed as methodologically weak.

There is no standard tool for data extraction or for assessing quality (Sanderson, Tatt, & Higgins, 2007). Guidelines such as the meta-analysis of observational studies in epidemiology (MOOSE) (Stroup et al., 2000) are designed for meta-analytic reviews. Examination of papers in this review indicated that a meta-analysis would not be appropriate. The Strengthening the Reporting of Observational studies in Epidemiology (STROBE) was therefore used. The STROBE statement was designed to guide data extraction from observational (von Elm et al., 2008) and cross-sectional study designs.

To ensure consistency in data extraction (Glasziou, Irwig, Bain, & Colditz, 2001), a data extraction template and a codebook were developed based on the STROBE (von Elm et al., 2008) and covered citation details, source of citation (e.g. CINAHL), study objectives, methods (selection of subjects, assessment, confounders and statistical analyses), results, conflict of interests and bias (Sanderson et al., 2007; von Elm et al., 2008). The template also included space for reviewers to make preliminary assessments of the information quality provided in the study (well covered, poor, adequate, not addressed, not reported or not applicable). Data extraction results were reviewed by all authors.

### 2.6. Synthesis of results

Results were first synthesised into a study summary (Table 2). Table 2 details the study methods, the outcome of interest, prevalence or mean of the outcome of interest, risk factors, prevalence or mean of the risk factor(s), the reported association between risk factor(s) and outcome of interest, and study strength. Second, significant risk factors were identified where Confidence Intervals did not overlap with 1 or where *r *= ±3 and are reported in Table 3.

**Table 2.** Summary of included studies examining factors associated with anxiety and depression in MDI.

Study	Study methods	Outcome(s)	Outcome prevalence or mean score (SD)	Risk factor(s)	Risk factor prevalence or mean score (SD)	Association between risk factor(s) and outcome(s)	Study strength	
							Level of evidence	Quality rating
Chen, Wong, and Yu (2009), China	*Study population*: offshore oil platform workers; *participant characteristics*: 100% male, mean age: 32.4 years; *sample size*: 561; *study design*: cross-sectional; *instrument*: GHQ −12; *analysis*: correlation	MH	10.2 (5.0)	Occupational stress	Not reported	*r* = 0.423, *p* < .001	IV	Weak
				Escaping/abreaction behaviours	Not reported	*r* = 0.221, *p* < .001		
				Internal behaviour	Not reported	*r* = 0.186, *p* < .001		
				Eating behaviour	Not reported	*r* = 0.029, *p* > .05		
				Positive attitude/denying behaviour	Not reported	*r* = 0.051, *p* > .05		
Cohidon, Santin, Imbernon, and Goldberg (2010), France	*Study population*: blue-collar worker sub-sample of a general population; *participant characteristics*: 52% male; *sample size*: 11,895 (pool: 16,848); *study design*: cross-sectional; *instrument*: CES-D; *analysis*: logistic regression (adjusted for social, demographic and health variables)	Depression	*Males*: 12.6%	Often go to bed after midnight	Not reported	*Males*: OR: 1.63, 95% CI: 1.01–2.65	IV	Moderate
			*Females*: 12.3%	Often awaken before 5a.m.	Not reported	*Males*: OR: 1.55, 95% CI: 1.02–2.36		
				Insufficient possibility of cooperation	Not reported	*Males*: OR: 2.05, 95% CI: 1.33–3.14		
				Often required to work fast (not bothered by it)	Not reported	*Females*: OR: 1.19, 95% CI: 0.41–3.40		
				Often required to work fast (bothered by it)	Not reported	*Females*: OR: 2.78, 95% CI: 1.08–7.18		
				Repetitive work	Not reported	*Females*: OR: 3.39, 95% CI: 1.63–7.05		
DeSanto Iennaco et al. (2010), US	*Study population*: heavy/aluminium industry workers; *participant characteristics*: 94% male, age range: 18–65 years, from 11 factories; *sample size*: 7566; *study design*: retrospective follow-up; *indicator*: insurance claims; *analysis*: logistic regression (adjusted for demographic and lifestyle variables)	Depression	4.6%	Demand	*High*: 5.7%	*High*: OR: 1.39, 95% CI: 1.04–1.86	III-2	Moderate
				*RC*: *Low demand*	*Moderate*: 4.7%	*Moderate*: OR: 1.33, 95% CI: 1.00–1.77		
					*Low*: 3.6%			
				Control	*High*: 4.2%	*Low*: OR: 0.78, 95% CI: 0.56–1.08		
				*RC*: *High control*	*Moderate*: 5.5%	*Moderate*: OR: 1.07, 95% CI: 0.81–1.43		
					*Low*: 4.0%			
				Gender	Males: 4.2%	*OR*: 2.41, 95% CI: 1.71–3.39		
				*RC*: *Males*	Females: 10.4%			
				Age	18–24 years: 4.4%	*18–25 yrs*: OR: 3.29, 95 CI%: 1.25–8.65		
				*RC*: *55–64 years*	25–34 years: 6.8%	*25–34 yrs*: OR: 4.92, 95% CI: 2.67–9.08		
					35–44 years: 6.4%	*35–44 yrs*: OR: 4.36, 95% CI: 2.55–7.46		
					45–54 years: 4.4%	*45–54 yrs*: OR: 3.11, 95% CI: 1.89–5.12		
					55–64 years: 1.4%			
d'Errico et al. (2011), Italy	*Study population*: blue-collar trade union members; *participant characteristics*: 77% male; *sample size*: 4,507 (response rate: 60% at baseline and 51% at follow-up); *study design*: prospective follow-up; *indicator*: prescriptions for anti-depressants; *analysis*: Poisson regression (adjusted for age, class, sex and occupational class)	Depression	Not reported	Shift work	Not reported	*2 shifts*: RR: 1.21, 95% CI: 0.86–1.70	II	Weak
				*RC*: *None*		*3–4 shifts*: RR: 1.01, 95% CI: 0.66–1.56		
						*Irregular shifts*: RR: 0.98, 95% CI: 0.42–2.27		
				Overtime	Not reported	≤ *4 h/week*: *RR*: 2.00, 95% CI: 1.02–3.92		
				*RC*: *None*		>*4 h/week*: RR: 1.08, 95% CI: 0.64–1.82		
				Excessive noise	Not reported	*Yes*: RR: 1.18, 95% CI: 0.86–1.63		
				*RC*: *No*				
				Psychological violence	Not reported	*Yes*: RR: 1.70, 95% CI: 1.03–2.80		
				*RC*: *No*				
				Demand	Not reported	*Intermediate*: RR: 1.34, 95% CI: 0.90–1.99		
				*RC*: *Low*		*High*: RR: 1.77, 95% CI: 1.20–2.62		
				Control	Not reported	*Intermediate*: RR: 0.73, 95% CI: 0.51–1.04		
				*RC*: *Low*		*High*: RR: 0.57, 95% CI: 0.33–0.97		
				Job strain	Not reported	*Intermediate*: RR: 1.05, 95% CI: 0.65–1.70		
				*RC*: *Low*		*High*: RR: 1.41, 95% CI: 0.91–2.19		
Ezoe and Morimoto (1994), Japan	*Study population*: manufacturing workers; *participant characteristics*: 76% male, age range: 20–59 years; *sample size*: 2,800 (response rate: 46.6%); *study design*: cross-sectional; *instrument*: GHQ-28 (anxiety and insomnia); *analysis*: multiple logistic regression (controlling for age, marital status and somatic condition)	Anxiety and insomnia	*Males*: Poor HPI: 41.6% Moderate HPI: 27.2% Good HPI: 17.9%*Females*: Poor HPI: 60.3% Moderate HPI: 37.3% Good HPI: 20.4%	Health trend during past 6 months	Not reported	*Males*: *B* = −0.468, *p* < .01 *Females*: *B* = 0.026, *p* < .05	IV	Weak
				Age	Not reported	*Males*: *B* = −0.006, *p* > .05		
				Mental stress	*Much*: 1.2 (1.4) *Average*: 0.9 (1.0) *Little*: 0.5 (0.8)	*Males*: *B* = 1.988, *p* < .001 *Females*: *B* = 1.908, *p* < .001		
				Nutritional balance	*Enough*: 0.5 (0.9) *A little*: 0.5 (1.0) *None*: 0.7 (1.2)	*Males*: *B* = −0.088, *p* > .05 *Females*: *B* = 0.076, *p* > .05		
				Breakfast	*Almost every day*: 0.5 (1.0) *Sometimes*: 0.7 (1.1) *Never*: 0.8 (1.2)	*Males*: *B* = −0.330, *p* < .05 *Females***:***B* = −0.334. *p* > .05		
				Physical exercise	*Twice or more p/wk*: 0.5 (1.0) *Once p/wk*: 0.6 (1.1) *Once or less p/month*: 0.5 (1.0) *Never*: 0.6 (1.1)	*Males*: *B* = −0.302, *p* > .05 *Females*: *B* = −0.026, *p* > .05		
				Sleeping hours per day	*9 or more*: 0.9 (1.6) 8: 0.6 (1.1) 7: 0.5 (1.0) 6: 0.6 (1.1) *5 or less*: 0.8 (1.1)	*Males*: *B* = −0.154, *p* > .05 *Females*: *B* = −0.138, *p* > .05		
				Alcohol consumption	*Almost every day*: 0.5 (1.1) *Sometimes*: 0.6 (1.0) *Never*: 0.6 (1.2)	*Males*: *B* = −0.074, *p* > .05 *Females*: *B* = 0.044, *p* > .05		
				Working hours per day	*11 or more*: 0.8 (1.3) 10: 0.6 (1.0) 9: 0.5 (0.9) *7 or less*: 1.0 (1.5)	*Males*: *B* = 0.006, *p* > .05 *Females*: *B* = 0.090, *p* > .05		
				Cigarettes	*Smoking*: 0.5 (1.0) *Quit*: 0.6 (1.0) *Never*: 0.5 (1.1)	*Males*: *B* = 0.110, *p* > .05 *Females*: *B* = −0.450, *p* > .05		
Inoue and Kawakami (2010), Japan	*Study population*: manufacturing workers; *participant characteristics*: 86% male, mean age: 37 years, from 9 factories; *sample size*: 25,104 (response rate range: 47–100%); *study design*: cross-sectional; *instrument*: CES-D; *analysis*: logistic regression (adjusted for age, marital status, overtime in the past month, chronic physical conditions, smoking status, drinking status, physical activity, supervisor support and co-worker support)	Depression	*Males*:	Interpersonal conflict	*Males (high SES)*:	*Males (high SES)*:	IV	Weak
			High SES: 20%	*RC*: *Low*	High: 37.6%	Moderate: OR: 2.68, 95% CI: 2.23–3.23		
			Moderate SES: 22.1%		Moderate: 21.6%	High: OR: 4.88, 95% CI: 4.04–5.90		
			Low SES: 26.8%		Low: 8.1%	*Males (moderate SES)*:		
			*Females*:		*Males (mod. SES)*:	Moderate: OR: 2.24, 95% CI: 1.77–2.83		
			High SES: 30.6%		High: 37.0%	High: OR: 4.09, 95% CI: 3.25–5.15		
			Moderate SES: 26.5%		Moderate: 21.1%	*Males (low SES)*:		
			Low SES: 31.5%		Low: 9.4%	Moderate: OR: 2.00, 95% CI: 1.71–2.33		
					*Males (low SES)*:	High: OR: 3.18, 95% CI: 2.73–3.70		
					High: 39.5%	*Females (high SES)*:		
					Moderate: 25.8%	Moderate: OR: 1.75, 95% CI: 1.04–2.94		
					Low: 13.4%	High: OR: 3.28, 95% CI: 1.89–5.69		
					*Females (high SES)*:	*Females (moderate SES)*:		
					High: 44.8%	Moderate: OR: 1.24, 95% CI: 0.81–1.90		
					Moderate: 30.7%	High: OR: 2.09, 95% CI: 1.40–3.13		
					Low: 20.1%	*Females (low SES)*:		
					*Females (mod. SES)*:	Moderate: OR: 1.80, 95% CI: 1.31–2.49		
					High: 36.2%	High: OR: 2.77, 95% CI: 2.03–3.78		
					Moderate: 24.3%			
					Low: 18.3%			
					*Females (low SES)*:			
					High: 42.7%			
					Moderate: 29.2%			
					Low: 17.6%			
Joensuu et al. (2010), Finland	*Study population*: forestry industry workers; *participant characteristics*: 75% male, mean age: 41.7 years; *sample size*: 13,868 (response rate: 62%); *study design*: prospective follow-up; *instrument*: ICD-9	Depression	*Depressive disorder*: 1.3%	Age	Not reported	*36–50 years*: HR 1.08, 95% CI: 0.79–1.48	II	Moderate
			HR: white-blue-collar: 1.55	*RC*: ≤ *35 years*		≥ *51 years*: HR 0.31, 95% CI 0.16–0.63		
				Gender	Not reported	*Male*: *HR*: 1.22, 95% CI: 0.69–1.72		
				*RC*: *Female*				
				Skill discretion	Not reported	*Intermediate*: HR: 0.67, 95% CI: 0.47–0.98		
				*RC*: *Low*		*High*: HR: 0.59, 95% CI: 0.37–0.92		
				Decision authority	Not reported	*Intermediate*: HR: 1.54, 95% CI: 1.06–2.25		
				*RC*: *Low*		*High*: HR: 1.70, 95% CI: 1.12–2.60		
				Supervisor support	Not reported	*Intermediate*: HR: 0.81, 0.57–1.17		
				*RC*: *Low*		*High*: HR: 0.90, 95% CI: 0.60–1.36		
				Co-worker support	Not reported	*Intermediate*: HR: 0.97, 95% CI: 0.67–1.41		
				*RC*: *Low*		*High***:** HR: 1.06, 95% CI: 0.72–1.57		
Kawada, Kuratomi, and Kanai (2009), Japan	*Study population*: manufacturing workers; *participant characteristics*: 100% male, aged 34–60 years; *Sample size*: 3,630; *Study design:* Cross-sectional; *instrument*: DSM-IV-TR; *analysis*: logistic regression	Depression	8.10%	Sleep	Sleep 6 hours or more: 39.2%	*>6 hours sleep*: OR: 0.44, 95% CI: 0.34–0.57, *p* < .01	IV	Weak
				*RC*: *<* *6 hours*				
				Age	34–39 years: 9.8%	OR: 0.98, 95% CI: 0.96–1.00		
				*RC*: *One year increments*	40–44 years: 8.8%			
					45–49 years: 7.1%			
					50–54 years: 8.0%			
					55–60 years: 4.5%			
Kawakami, Haratani, and Araki (1992), Japan	*Study population*: machine operators, assemblers, production inspectors and mechanics in an electrical factory; *participant characteristics*: 100% male, age range 20–49 years; *sample size*: 468 (response rate: 37%); *study design*: prospective follow-up; *instrument*: Zung Self-Rated Depression Scale; *analysis*: binomial regression (controlling for baseline depression, age, marital status, education, medical treatment and type A behaviour)	Depression	*Time 0*: 13%	Lack of control over workplace	Not reported	*Time 1*: RR: 1.71, 95% CI: 1.10–2.65	II	Moderate
			*Time 1*: 10.5%	*RC*: *Lower*				
			*Time 2*: 9.8%	Job unsuitability	Not reported	*Time 2*: RR: 1.85, 95% CI: 1.28–2.68		
			*Time 3*: 11.3%	*RC*: *Lower*		*Time 3*: RR: 1.94, 95% CI: 1.08–3.46		
				Poor human relations at workplace	Not reported	*Time 2*: RR: 1.94, 95% CI: 1.17–3.20		
				*RC*: *Lower*				
Kleppa, Sanne, and Tell (2008), Norway	*Study population*: general population; *participant characteristics*: males and females born between 1953–1957 who worked ≥ two hours per week; *sample size*: 10,442; *study design*: case-control target sampling; *instrument*: HADS-A & HADS-D; *analysis*: logistic regression (unadjusted)	Anxiety	*Low-skill workers*^a^:	Occupational group	Not reported	*Males*:	III-2	Moderate
			Males:	*RC*: *High-skill workers*^b^		Intermediate-skill workers^c^:		
			15.6%; Mean: 4.46;			OR: 1.07, 95% CI: 0.86–1.34		
			95% CI: 4.33–4.60			Low-skill workers:		
			Females:			OR: 1.19, 95% CI: 1.01–1.39		
			23.1%; *M* = 5.18;			*Females*:		
			95% CI: 4.95–5.42			Intermediate-skill workers:		
						OR: 1.01, 95% CI: 0.85–1.19		
						Low-skill workers:		
						OR: 1.11, 95% CI: 0.84–1.46		
				Level of physical activity at work	Not reported	*Males*:		
				*RC*: *Mainly sedentary work*		Much walking ± much lifting:		
						OR: 1.06, 95% CI: 0.92–1.24		
						Heavy manual labour:		
						OR: 1.25, 95% CI: 0.96–1.65		
						*Females*:		
						Much walking ± much lifting:		
						OR: 1.01, 95% CI: 0.89–1.15		
						Heavy manual labour:		
						OR: 1.32, 95% CI: 0.65–2.69		
		Depression	*Low-skill workers*:	Occupational group	Not reported	*Males*:		
			Males:	*RC*: *High-skill workers*		Intermediate-skill workers:		
			12.9%; Mean: 3.92			OR: 1.31, 95% CI: 1.00–1.71		
			95% CI: 3.79–4.05			Low-skill workers:		
			Females:			OR: 1.76, 95% CI: 1.46–2.11		
			10.6%; Mean: 3.41			*Females*:		
			95% CI: 3.20–3.61			Intermediate-skill workers:		
						OR: 1.18, 95% CI: 0.91–1.52		
						Low-skill workers:		
						OR: 1.72, 95% CI: 1.18–2.50		
				Level of physical activity at work	Not reported	*Males*:		
				*RC*: *Mainly sedentary work*		Much walking ± much lifting:		
						OR: 1.17, 95% CI: 0.98–1.40		
						Heavy manual labour:		
						OR:1.49, 95% CI: 1.10–2.02		
						*Females*:		
						Much walking ± much lifting:		
						OR: 1.04, 95% CI: 0.86–1.25		
						Heavy manual labour:		
						OR: 1.63, 95% CI: 0.64–4.18		
Maffeo et al. (1990), US	*Study population*: nuclear industry job seekers; *participant characteristics*: 79% male, 87% aged 20–50 years; *sample size*: 2,290; *study design*: cross-sectional; *instrument*: MMPI-D; *analysis*: generalised linear modelling	Depression	*Males*: 52.70 (8.64) *Females*: 47.90 (7.11)	Occupation Level (schedule)	Least square means on D30 T-scores (subset of MMPI-D)	F(8,2280) = 3.79, *p* < .001	IV	Weak
					High level mgrs: 41.323			
					Low level mgrs: 43.169			
					Operations personnel: 43.379			
			No overall significant differences on depression scores according to gender		Professional: 44.049			
					Administrative-technical: 44.348			
					Public safety: 44.684			
					Trades and labour: 46.583			
					Clerical: 47.148			
					Janitorial: 48.534			
McShane and Quirk (2009), Australia	*Study population*: farmers; *participant characteristics*: 90% male, age range 30–81 years; *sample size*: 50; *study design*: cross-sectional; *instrument*: DASS – Anxiety & DASS – Depression; *analysis*: multiple regression	Anxiety	6.82 (9.88)	Personal finance	Not reported	Mediating effect: *B* = 2.80, *p* < .05	IV	Weak
				Work–home interference	Not reported	Mediating effect: *B* = 7.15, *p* < .001		
				Time pressure –work–home interference	Not reported	Mediating effect: *B* = 3.80, *p* < .05		
				Strain-work–home interference	Not reported	Mediating effect: *B* = 3.94, *p* < .05		
		Depression	9.76 (10.43)	Personal finance	Not reported	Mediating effect: *B* = 2.87, *p* < .05		
				Work–home interference	Not reported	Mediating effect: *B* = 8.17, *p* < .01		
				Time pressure –work–home interference	Not reported	Mediating effect: *B* = 3.84, *p* < .05		
				Strain-work–home interference	Not reported	Mediating effect: *B* = 4.44, *p* < .05		
Niedhammer et al. (1998), France	*Study population*: electrical company employees; *participant characteristics*: men age range: 46–56 years and women age range: 41–56 years; *sample size*: 11,552; *study design*: prospective cohort; *instrument*: CES-D; *analysis*: adjusted logistic regression	Depression	*Males*: 24.9%	Stressful occupational events	*Males*:	*Males*:	II	Moderate
			*Females*: 27.9%	*RC*: *No events*	0: 22.3%	1 event: OR: 1.57, 95% CI: 1.37–1.79		
					1: 31.3%	≥2 events: OR:1.73, 95% CI: 1.40–2.14		
					≥2: 31.7%	*Females*:		
					*Females*:	1 event: OR: 1.44, 95% CI: 1.14–1.82		
					0: 24.8%	≥2 events: OR: 2.04, 95% CI: 1.47–2.85		
					1: 33.6%			
					≥2: 41.4%			
				Psychological demands	*Males*:	*Males*: OR: 1.77, 95% CI: 1.57–1.99		
				*RC*: *Low*	*Low*: 20.2%	*Females*: OR: 1.37, 95% CI: 1.13–1.67		
					High:30.5%			
					*Females*:			
					Low: 24.4%			
					High:30.1%			
				Decision latitude	*Males***:**	*Males*: OR: 1.38, 95% CI: 1.22–1.56		
				*RC*: *High*	High: 21.3%	*Females*: OR: 1.41, 95% CI: 1.15–1.73		
					Low: 28.7%			
					*Females*:			
					High: 23.0%			
					Low: 32.2%			
				Social support at work	*Males*:	*Males*: OR: 1.58, 95% CI: 1.41–1.78		
				*RC*: *High*	High: 19.7%	*Females*: OR: 1.29, 95% CI: 1.06–1.57		
					Low: 29.09%			
					*Females*:			
					High: 24.8%			
					Low: 30.4%			
				Stressful personal events	*Males*:	*Males*:		
				*RC*: *No events*	0: 22.4%	1 event: OR: 1.15, 95% CI: 1.01–1.31		
					1: 25.2%	2 events: OR: 1.58, 95% CI: 1.33–1.87		
					2:30.8%	≥3 events: OR: 1.77, 95% CI: 1.32–2.37		
					≥3: 33.1%	*Females*:		
					*Females*:	1 event: OR: 1.53, 95% CI: 1.23–1.90		
					0: 22.2%	2 events: OR: 2.02, 95% CI: 1.52–2.69		
					1: 30.0%	≥3 events: OR: 3.17, 95% CI: 2.08–4.82		
					2: 34.8%			
					≥3: 49.2%			
Niedhammer et al. (2006), France	*Study population*: general population of workers and 150 employees of occupational physicians; *participant characteristics*: 69% male, mean age: 40 years; *sample size*: 7,694; *study design*: cross-sectional; *instrument*: CES-D; *analysis*: logistic regression (adjusted for age, marital status, presence of children, education & occupation)	Depression	*Whole sample*:	Exposure to bullying	*Males*: 68.63%	*Males*: OR: 8.00, 95% CI: 6.06–10.56	IV	Moderate
			Males: 25.43%	*RC*: *No exposure to bullying*	*Females*: 60.63%	*Females*: OR: 8.44, 95% CI: 6.84–10.41		
			Females: 21.18%					
			*Blue-collar workers*:					
			*Males*: 27.81%					
			*Females*: 28.74%					
Oldfield and Mostert (2007), Sth Africa	*Study population*: miners; *participant characteristics*: 80% male, age range: 30–49 years; *sample size*: 320; *study design*: cross-sectional; *instrument*: GHQ; *analysis*: correlation	Anxiety and Insomnia	12.96 (4.68)	Pressure	Not reported	*r* = 0.17, *p* <.05	IV	Weak
				Poor work conditions	Not reported	*r* = 0.23, *p* < .05		
				Autonomy	Not reported	*r* = −0.15, *p* < .05		
				Task characteristics	Not reported	*r* = −0.15, *p* < .05		
				Social support	Not reported	*r* = −0.22, *p* < .05		
				Instrumental support	Not reported	*r* = −0.15, *p* < .05		
				Pay and benefits	Not reported	*r* = −0.05, *p* > .05		
				Somatic complaints	Not reported	*r* = 0.67, *p* < .05		
				Exhaustion	Not reported	*r* = 0.38, *p* < .05		
				Negative WHI	Not reported	*r* = 0.38, *p* < .05		
Rose, Beh, Uli, and Idris (2006), Sweden	*Study population*: automotive industry workers; *participant characteristics*: 100% male, born between 1943–1948; *sample size*: 954; *study design*: prospective follow-up; *instrument*: PGWB; *analysis*: unadjusted multivariate regression	Anxiety	*Blue-collar workers*:	Age	Not reported	*r* = 0.20, *p* < .05	II	Strong
			25.4 (3.95)	Occupational category	Not reported	*r* = −0.51, *p* < .05		
			*White-collar workers*:	Job satisfaction	Not reported	*r* = 0.02, *p* > .05		
			24.7 (3.89)	Support	Not reported	*r* = 0.13, *p* > .05		
				Frequency of feelings of nervousness	Not reported	*r* = −0.73, *p* < .001		
				Frequency of feelings of depression	Not reported	*r* = −0.47, *p* < .001		
				Work-related life events	Not reported	*r* = −0.58, *p* < .05		
		Depression	*Blue-collar workers*:	Age	Not reported	*r* = 0.25, *p* < .001		
			16.6 (3.21)	Occupational category	Not reported	*r* = 0.19, *p* > .05		
			*White-collar workers*:	Support	Not reported	*r* = 0.08, *p* < .01		
			16.7 (1.81)	Frequency of feelings of nervousness	Not reported	*r* = −0.35, *p* < .001		
				Frequency of feelings of depression	Not reported	*r* = −0.16, *p* < .001		
Rusli, Edimansyah, and Naing (2008), Malaysia	*Study population*: petroleum and automobile assembly plant employees; *participant characteristics*: 100% male, mean age: 27 years (SD 5.9); *sample size*: 691; *study design*: cross-sectional; *instrument*: DASS-Anxiety & DASS-Depression; *analysis*: correlation	Anxiety	8.3 (5.5)	Age	Not reported	*r* = 0.13, *p* < .01	IV	Weak
				Job demand	Not reported	*r* = 0.18, *p* < .01		
				Job control	Not reported	*r* = 0.04, *p* > .05		
				Social support	Not reported	*r* = −0.14, *p* < .01		
				Stress	Not reported	*r* = −0.79, *p* < .01		
				Depression	Not reported	*r* = 0.74, *p* < .01		
				Physical health	Not reported	*r* = −0.40, *p* < .01		
				Psychological status	Not reported	*r* = −0.19, *p* < .01		
				Environment	Not reported	*r* = −0.27, *p* < .01		
				Social relationships	Not reported	*r* = −0.23, *p* < .01		
		Depression	8.3 (5.8)	Age	Not reported	*r* = 0.12, *p* < .01		
				Job demand	Not reported	*r* = 0.19, *p* < .01		
				Job control	Not reported	*r* = −0.03, *p* > .05		
				Social support	Not reported	*r* = −0.23, *p* < .01		
				Stress	Not reported	*r* = 0.84, *p* < .01		
				Anxiety	Not reported	*r* = 0.74, *p* < .01		
				Physical health	Not reported	*r* = −0.39, *p* < .01		
				Psychological status	Not reported	*r* = −0.27, *p* < .01		
				Environment	Not reported	*r* = −0.33, *p* < .01		
				Social relationships	Not reported	*r* = −0.29, *p* < .01		
Savikko, Lanne, Spak, and Hensing (2008), Sweden	*Study population*: longitudinal cohort from the Women and Alcohol in Göteborg Study; *participant characteristics*: 100% female; *sample size*: 562; *study design*: cross-sectional; *instrument*: DSM-III-R; *analysis*: regression (adjusted for age, having dependent children, & early background factors)	Anxiety (Shorter duration or minor severity)	Not reported	Proportion of females in occupation	Not reported	*0–20%*: OR: 0.7, 95% CI: 0.2–2.4	IV	Weak
				*RC*: *41–60%*		*21–40%*: OR: 0.8, 95% CI: 0.3–2.1		
						*61–80%*: OR: 0.8, 95% CI: 0.4–1.8		
						*81–100%* OR: 0.6, 95% CI: 0.3–1.3		
		Anxiety (Longer duration or higher severity)	Not reported	Proportion of females in occupation	Not reported	*0–20%*: OR: 0.1, 95% CI: 0.0–1.0		
				*RC*: *41–60%*		*21–40%*: OR: 0.7, 95% CI: 0.3–1.9		
						*61–80%*: OR: 0.4, 95% CI: 0.2–0.99		
						*81–100%*: OR: 0.5, 95% CI: 0.3–1.1		
Scarth, Stallones, Zwerling, and Burmeister (2000), US	*Study population*: farmers; *participant characteristics*: 100% male, mean age: 50.1 years; *sample size*: 855; *study design*: cross-sectional; *instrument*: CES-D; *analysis*: logistic regression	Depression	9.8%;	Legal problems	Yes: 7.5%	*OR*: 4.67, 95% CI: 2.39–9.13	IV	Weak
			Mean: 6.24 (6.99);	*RC*: *No*				
			Range: 0–53	Marital status	Unmarried: 9.0%	*OR*: 3.67, 95% CI: 1.53–7.83		
				*RC*: *Married*				
				Sentimental value loss	Yes: 17.1%	*OR*: 3.20, 95% CI: 1.64–6.24		
				*RC*: *No*				
				Substantial income decrease	Yes: 31.8%	*OR*: 2.71, 95% CI: 1.59–4.63		
				*RC*: *No*				
				General health assessment	*Excellent*: 26.2%	*Very good***:** OR: 1.94, 95% CI: 0.65–5.83		
				*RC*: *Excellent*	*Very good*: 40.0%	*Good*: OR: 3.60, 95% CI: 1.50–8.62		
					*Good*: 26.3%	*Fair/Poor*: OR: 6.79, 95% CI: 2.51–18.38		
					*Fair*: 6.2%			
					*Poor*: 1.4%			

Notes: M: mean, SES: socio-economic status, HPI: health practice index, WHI: work–home interference, MH: mental health, RR: relative risk, HR: hazard ratio, OR: odds ratio, CI: confidence interval, RC: reference category.

^a^Agriculture/forestry/fishery workers, craft and related trades workers, plan/machine operators, assemblers, and elementary occupations.

^b^Armed forces, legislators/senior officials/managers, professionals, technicians/associate professionals.

^c^Clerks, shop/market sales and service workers.

**Table 3.** Summary of factors associated^a^ with anxiety and depression.

Risk factor	Number of studies	Risk associations
*Individual factors*		
Age	2	• Younger and mid-age groups more depressed (DeSanto Iennaco et al., 2010), older group less depressed (over 51 years) (Joensuu et al., 2010)
Gender	2	• Women more likely to have depression than men (DeSanto Iennaco et al., 2010)
		• Higher proportion of women in MDI associated with less severe anxiety for women (Savikko et al., 2008)
Health	7	• Poorer self-reported general health (Rusli et al., 2008; Scarth et al., 2000); during past six months for males (Ezoe & Morimoto, 1994)
		• Stress, depression, anxiety (Rusli et al., 2008), mental stress (Ezoe & Morimoto, 1994)
		• Sleep patterns: often go to bed after midnight and wake before 5 a.m.(Cohidon et al., 2010), less than six hours sleep a night (Kawada et al., 2009)
		• Not eating breakfast for males (Ezoe & Morimoto, 1994)
		• Somatic complaints, exhaustion (Oldfield & Mostert, 2007)
		• Reporting feelings of nervousness and depression strongly correlated with anxiety and depression (Rose et al., 2006)
Life events	3	• Stressful personal events in past 12 months (Niedhammer et al., 1998)
		• Legal problems or sentimental loss in past 12 months (Scarth et al., 2000)
		• Substantial income decrease in previous 12 months (Scarth et al., 2000), personal finance (loss of income) (McShane & Quirk, 2009)
Marital status	1	• Being unmarried (Scarth et al., 2000)
*Team environment*		
Workplace relationships	6	• Psychological violence (d’Errico et al., 2011)
		• Workplace bullying (Niedhammer et al., 2006)
		• Low levels of social support at work (Neidhammer et al., 1998)
		• Absence of workplace cooperation (Cohidon et al., 2010)
		• Poor human relations at workplace (Kawakami et al., 1992) and interpersonal conflict (Inoue & Kawakami, 2010)
*Work conditions*		
Job suitability and skill discretion	2	• Job unsuitability (Kawakami et al., 1992)
		• Low-skill discretion (Joensuu et al., 2010)
Occupational/salary level	4	• Lower skilled occupations, lower occupational levels and blue-collar work were associated with more anxiety and depression (Kleppa et al., 2008; Maffeo et al., 1990; Rose et al., 2006)
		• Higher occupational levels had lower levels of depression (Maffeo et al., 1990)
Job control	4	• Lack of control over workplace (d'Errico et al., 2011; Kawakami et al., 1992)
		• Low levels of decision latitude (Niedhammer et al., 1998) and high decision authority (Joensuu et al., 2010)
Job overload and job demands	4	• Often required to work fast (and bothered by it), without error, with conflicting demands; time pressures, need to constantly concentrate, repetitive work, high and intermediate demand work, high job strain, job overload (Cohidon et al., 2010; d’Errico et al., 2011; DeSanto Iennacco et al., 2010)
		• Working atypical hours (Cohidon et al., 2010) and overtime (d’Errico et al., 2011)
		• High level of psychological demands (Niedhammer et al., 1998)
Occupational stress and work changes	4	• Non-specific occupational stress (Chen et al., 2009)
		• Stressful occupational events, particularly for females (Niedhammer et al., 1998)
		• Negative work-related life events (e.g. business readjustment, change to a different line of work, change in responsibilities at work or change in working hours and conditions), especially for blue-collar workers (Rose et al., 2006)
		• Environmental conditions (Rusli et al., 2008)
*Work–Home interference*		
WHI interference	2	• Time pressure, work strain and conflict between work demands and family roles (e.g. McShane & Quirk, 2009; Oldfield & Mostert, 2007)

^a^Risk factors were included if Confidence Intervals do not overlap with 1 or where *r* = ±0.3.

## 3. Results

Nineteen studies from a variety of MDI and a range of countries and cultures met the inclusion criteria. The prevalence rates and scores on measures for anxiety and depression in MDI differed according to country, industry type, occupational category and scale used for measurement, as well as a range of other variables (see Table 2).

### 3.1. Risk factor domains

Four groups of risk factors associated with anxiety and depression were identified: individual factors (life events, job fit and demographic factors); team environment (workplace relationships); work conditions (job demand, job variety, job control); and work–home interactions (see Table 3). Many of these factors were mediated by personal, demographic and role characteristics.

#### 3.1.1. Individual factors

Individual risk factors associated with anxiety and depression included negative and stressful life events in the past year (e.g. legal or financial problems) (McShane & Quirk, 2009; Niedhammer, Goldberg, Leclerc, Bugel, & David, 1998; Scarth et al., 2000). Poorer physical health (Ezoe & Morimoto, 1994; Rusli et al., 2008; Scarth et al., 2000), lack of sleep (Cohidon et al., 2010; Kawada et al., 2009), mental stress and exhaustion (Ezoe & Morimoto, 1994; Oldfield & Mostert, 2007; Rusli et al., 2008), not eating breakfast (Ezoe & Morimoto, 1994) and marital status (Scarth et al., 2000) were also associated with anxiety and depression. Older age was generally a protective factor (DeSanto Iennaco et al., 2010; Joensuu et al., 2010).

Six studies had samples consisting only of males, 9 had samples consisting of 75 + % male participants, 1 was of females only and 3 had both male and female participants. DeSanto Iennaco et al. (2010) found that women were significantly more likely to have depression compared with their male colleagues, although the presence of a higher proportion of women in MDI was associated with less severe anxiety for women (Savikko et al., 2008).

#### 3.1.2. Team environment

Interpersonal conflict, poor cooperation and workplace relationships, and lack of support at work were risk factors for depression (Cohidon et al., 2010; d'Errico et al., 2011; Inoue & Kawakami, 2010; Kawakami et al., 1992; Niedhammer, David, & Degioanni, 2006; Niedhammer et al., 1998). The impact of interpersonal conflict upon anxiety and depression appeared to be mediated by other factors, such as socio-economic status (SES) and gender (Inoue & Kawakami, 2010). Both men (OR: 8.00, 95% CI: 6.06–10.56) and women (OR: 8.44, 95% CI: 6.84–10.41) who had ever been bullied, or who had witnessed the bullying of others, were significantly more likely to be depressed than those who had not been exposed to bullying (Niedhammer et al., 2006).

#### 3.1.3. Work conditions

Lower skilled occupations, lower occupational levels and blue-collar workers were associated with more anxiety and/or depression (Kleppa et al., 2008; Maffeo et al., 1990; Rose et al., 2006). The Maffeo et al. (1990) study found that higher level managers had significantly fewer symptoms of depression than other occupation categories including trade and labour.

*Job demand*. Job overload and high job demand (work requiring high levels of physical or mental effort; fast-paced and repetitive work) were associated with poorer MH (Cohidon et al., 2010; d'Errico et al., 2011; DeSanto Iennaco et al., 2010; Kleppa et al., 2008; Niedhammer et al., 1998). Increased job demand also increased risk of depression, even after controlling for demographic and lifestyle factors (OR: 1.39, 95% CI: 1.04–1.86) (DeSanto Iennaco et al., 2010). Excessive overtime was also associated with depression (Cohidon et al., 2010; d'Errico et al., 2011). Higher scores for anxiety were also associated with male low-skill workers (Kleppa et al., 2008).

High demand was found to significantly increase risk of depression among blue-collar workers (RR = 1.77) (d'Errico et al., 2011). In Niedhammer et al. (1998) prospective study, high psychological demand was a risk factor for depression in both men (OR: 1.77, 95% CI: 1.57–1.99) and women (OR: 1.37, 95% CI: 1.13–1.67). Excessive overtime (d'Errico et al., 2011) and the requirement to work fast (Cohidon et al., 2010) also increased depression among blue-collar workers.

Other factors associated with anxiety and depression included work changes (e.g. business readjustment) (Rose et al., 2006) and occupational stressors (Chen et al., 2009; Niedhammer et al., 1998). Anxiety was significantly associated with negative work-related events (such as business readjustment, changes in working hours and conditions), which affected blue-collar workers more adversely than white-collar workers (Rose et al., 2006). Broader environmental conditions were also associated with depression (Rusli et al., 2008).

*Job control.* Lack of job control (d'Errico et al., 2011; Kawakami et al., 1992) was associated with poorer MH outcomes. However, the effect of job control on MH was mediated by demographic and job variables.

Niedhammer et al. (1998) found an increased risk of depression when there was low decision latitude (compared with high decision latitude) for both men (OR: 1.38, 95% CI: 1.22–1.56) and women (OR: 1.41, 95% CI: 1.15–1.73). However, the ability to make decisions about one's own job and influence the work team was also associated with increased risk for depression (HR: 1.70, 95% CI: 1.12–2.60) (Joensuu et al., 2010).

Job unsuitability, or poor job fit, was also significantly associated with depression (Kawakami et al., 1992). More opportunity to use one's skills was significantly associated with reduced MH disorders (HR 0.74, 95% CI: 0.58–0.95) including depression (HR 0.59, 95% CI: 0.37–0.92) (Joensuu et al., 2010).

#### 3.1.4. Work–home interference

Work–home interference (the influence of work performance on home life) was associated with MH problems. Among farmers, work stressors including time pressures and work strain interfered with home life and increased farmers’ reported psychological distress (McShane & Quirk, 2009). Work to home stressors were more salient than home to work stressors (McShane & Quirk, 2009). Another study found that job demands and few job resources (including autonomy, task characteristics, social support at work, technical support at work and pay and benefits) were associated with anxiety and insomnia, and in turn, negative work–home interference (i.e. work negatively influenced home life) (Oldfield & Mostert, 2007).

## 4. Discussion

This systematic review examined risk factors for anxiety and depression among workers in MDI. Previous reviews have considered risk factors for anxiety and depression in the workplace, but none has specifically focused on MDI, despite workers in these industries showing higher than average rates of anxiety and mood disorders (Australian Bureau of Statistics, 2008b). Nineteen studies were identified that met the inclusion criteria.

The findings are generally consistent with earlier systematic reviews that have identified the risk factors associated with poorer MH outcomes amongst workers (Michie & Williams, 2003; Stansfeld & Candy, 2006). This study found a range of anxiety and depression risk factors in MDI, which were categorised into individual factors, team environment, work conditions and work–home interference. Work conditions and team environment were most commonly identified. In particular, unsupportive workplace relationships, job overload and job demands were risk factors for anxiety and depression. These factors were mediated by job status (e.g. blue-/white-collar work). There was also a moderate level of evidence that individual factors such as health status and life events influence anxiety and depression. There was also some evidence for the influence of work–home interference on MH.

### 4.1. Comparison with previous studies

The job demand-control model and its offshoot, the job control-demand-support model, are organisational approaches that have been used since the 1980s. They posit that jobs with high demand, low control (decision latitude) and low social support contribute to low psychological well-being and poor physical health (Amagasa & Nakayama, 2012; Karasek et al., 1988; Kristensen, 1995; Marmot, Siegrist, & Theorell, 1999; Stansfeld & Candy, 2006). In particular, the combinations of high job demands and low decision latitude (job strain), and high effort and low rewards (reward-effort imbalance) are found to be risk factors for mental disorders, emphasising the importance of the psychological work environment (Stansfeld & Candy, 2006).

The findings in this study regarding the importance of ‘work conditions’ were generally consistent with Stansfield and Candy's (2006) earlier meta-analytic review. Stansfield and Candy's (2006) study identified workplace psychosocial environment, including job strain, low decision latitude, psychological demands, low social support, high job insecurity and effort–reward imbalance, as predictors of common mental disorders, with job strain and effort–reward imbalance having the strongest effects on MH. However, the risk factor ‘reward-effort imbalance’ (where effort expended is perceived to exceed job rewards), identified by Stansfeld and Candy (2006) as an important factor in predicting MH problems, was not highlighted as an important risk factor for MH problems in MDI in the present study – studies included in the review were less focused on this aspect of work.

The present review also found that blue- and white-collar workers differentially experienced various types of risk factors. Blue-collar workers with high demand work experienced more depression than white-collar workers with similar demands (d’Errico et al., 2011), and had more anxiety as a result of exposure to risks such as negative work-related life events than white-collar workers (Rose et al., 2006). In d'Errico et al.’s (2011) study, high demand was a protective factor for white-collar workers only. This difference between blue- and white-collar workers with regard to the impact of ‘high demand’ work may potentially be explained by other mediating factors such as ‘skill discretion’, which is a protective factor for mental disorders, and is often more strongly associated with white-collar jobs (Joensuu et al., 2010).

However, results for job control were mixed. In one study of an industrial cohort, low job control was not associated with depression (DeSanto Iennaco et al., 2010). In Joensuu et al.’s (2010) study, high decision authority was associated with increased risk of depression for both blue- and white-collar industrial employees. Our findings suggest that stress for blue-collar workers may increase with both high decision authority (the ability to influence one's own and others' work) and high psychological demand. This finding is slightly different from that of previous studies, where a combination of low decision latitude and high demands was associated with poorer MH (e.g. Stansfeld & Candy, 2006). This finding may be related to the different nature of work/responsibilities undertaken by blue-collar workers or the workplace culture where blue-collar workers become decision-makers. This issue is an avenue for future exploration. Blue-collar workers may need additional support in situations where they are expected to make decisions or where job demands increase (e.g. after being promoted to a leadership position or allocated extra responsibilities).

Many studies, one of which involved blue-collar workers only (Kawakami et al., 1992), established poor human relations and lack of support or cooperation at work as a risk factor for mental disorders (Cohidon et al., 2010; d’Errico et al., 2011; Inoue & Kawakami, 2010; Kawakami et al., 1992; Niedhammer et al., 2006). One study found a significant association between depression and interpersonal conflict (Inoue & Kawakami, 2010). This effect was particularly mediated by SES (Inoue & Kawakami, 2010). There was a very strong association between workplace bullying and depression (Niedhammer et al., 2006).

This review highlights the importance of job factors such as work overload/job demands, whilst negative work-related events (such as business readjustment, changes in working conditions and hours, changes in work responsibilities) appeared to affect the MH of blue-collar workers more than white-collar workers. However, a study on work-related risk factors for anxiety and depression, by Nydegger (2002), found that organisational factors (e.g. changes in technology, physical working conditions, management styles and attitudes, and structure of organisations) are more salient work stressors than job factors (e.g. role conflict, role ambiguity, responsibility for others, work underload and overload and harassment/sexual harassment).

### 4.2. Implications

Opportunities for primary and secondary prevention of MH problems are of paramount importance (Barry, Canavan, Clarke, Dempsey, & O'Sullivan, 2009). Consistent with a ‘healthy settings’ approach to health promotion, the workplace holds considerable promise as a setting in which to introduce strategies that can prevent and/or ameliorate MH problems among a largely difficult-to-access population.

Few studies have identified the intervention strategies specifically for MDI, but several have examined the interventions for MH in the workplace more generally (Barry et al., 2009; Cooper & Cartwright, 1994; Giga et al., 2003; LaMontagne, Keegel, Louie, Ostry, & Landsbergis, 2007). Historically, MH-related workplace interventions have targeted the individual worker with varying degrees of success and inconclusive long-term outcomes (Cooper & Cartwright, 1994, 1997; van der Klink, Blonk, Schene, & Van Dijk, 2003). Alternatively, primary prevention through ‘proactive’ organisation-directed activities (e.g. increased social support and job control in the workplace), to circumvent the need for ‘reactive’ secondary (e.g. stress management) and tertiary (e.g., Employee Assistance Programmes) prevention, has been suggested (Cooper & Cartwright, 1994).

Most workers have relatively little control over workplace factors. However, there is considerable latitude at the organisational and managerial levels to address crucial factors that impact workers' MH and well-being. By addressing issues of social support and the team environment, job demand, job variety and job control, workplaces can have a positive primary and secondary preventive influence on the MH and well-being of employees. This review has highlighted specific areas of risk where workplace programmes have potential to prevent and/or ameliorate mental problems among workers.

Although individually focused approaches affect the individual-level outcomes, they do not influence organisational level change (LaMontagne et al., 2007). By contrast, organisational approaches have benefits at both individual and organisational levels, and offer a greater scope for the prevention of MH problems in MDI (Cooper & Cartwright, 1994).

Organisational approaches to workplace MH promotion and prevention could include supervisory and psychological support for staff, enhanced job control, increased staff involvement in decision-making, workload assessment, effort/reward balance, role clarity and policies to reduce bullying and harassment (Cooper & Cartwright, 1994; Keleher & Armstrong, 2005; World Health Organization, 2005).

Growing emphasis has been placed on the duty of care of employers towards their employees through occupational health and safety (OHS) law and policy, including the duty to provide a safe workplace to promote both physical and psychological health. OHS has traditionally focused on physical safety. There is increasing recognition of the need for ‘psychologically safe’ work environments (Dollard & Bakker, 2010).

The present review highlights the scope for a primary prevention focus on blue-collar workers through organisational measures. Blue-collar workers, compared with white-collar workers, experienced more anxiety and depression and were differentially affected by or more exposed to job-related factors associated with depression and anxiety. They are often located in lower SES jobs commonly associated with risk factors for MH problems, including repetitive work, low-skill discretion and higher job insecurity (Borg & Kristensen, 2000; Kristensen, Borg, & Hannerz, 2002). Atypical hours and excessive overtime negatively impact blue-collar workers' MH. Overtime was also a risk factor for depression among white-collar workers, as was lack of job control. Inclusive decision-making, increased autonomy and input into the workplace may help protect these workers from depression and anxiety. Another important factor to consider is other non-work-related MH risk factors (such as negative life events) (Rose et al., 2006). Due to the inter-relationship between the determinants of health, workers in blue-collar jobs are more likely to experience other determinants of poor health (e.g. negative life events due to the relationship between SES, health, insecure housing and low education).

Research into worker health also highlights the changing nature of the world of work, including the impact of globalisation (Dollard & Bakker, 2010). Such changes have included technological advances, longer working days, dual-income families, increased workloads, decreased job security and greater home–work interference, balancing work and non-work commitments (Barry et al., 2009; Dollard & Bakker, 2010; O'Driscoll, Brough, & Biggs, 2007) and more frequent job restructuring and contractual work (Barry et al., 2009). Technological advances (e.g. email, smart phones) mean that people are never fully away from work, blurring distinctions between work and home life, and potentially leading to increased job demands (Pollett, 2007). Economic constraints can also lead to a phenomenon of over-employment with cuts to workforce numbers despite high workloads (Dollard & Winefield, 2002).

Blue-collar workers are most likely to be affected by restructuring and organisational downsizing, resulting in considerable psychological distress (Eurofound, 2012; Parker, Chmiel, & Wall, 1997), which may be ameliorated through appropriate consultation, increased worker control over their day-to-day functions and participation in downsizing processes (Parker et al., 1997). In such instances, re-employment programmes and tertiary prevention (Cooper & Cartwright, 1994) through access to employee assistance programmes may be beneficial.

### 4.3. Limitations and future studies

Although few studies examined differences across industries and most were cross-sectional in nature (rather than prospective studies), limiting the potential generalizability of the findings, the results of this body of research overall were relatively consistent. However, one confounding factor in this study may be the relationship between alcohol and MH problems. The relationship between risky drinking and MDI was in fact part of the wider systematic review (Roche et al., 2012) and will be reported elsewhere. As anxiety and depression correlate with other MH disorders, an examination of psychosocial work factors and other MH problems is likely to show similar findings.

Variations in the study samples are noted, particularly in relation to culture/country, gender and occupational categories. Six studies had male-only samples, and one had a female-only sample. A range of measures were also used to assess anxiety and depression (e.g. hospital admissions, insurance claims, tools used in clinical settings and the GHQ). This level of heterogeneity precluded a meta-analysis.

More research is needed that employs reliable clinical measures and utilises longitudinal and randomised controlled trial study designs (Caulfield, Chang, Dollard, & Elshaug, 2004; Michie & Williams, 2003; Murphy & Sauter, 2004; Semmer, 2004). It is also incumbent upon researchers, clinicians and practitioners to effectively disseminate evidence-based strategies to ensure that workplaces have the capacity and motivation to address issues that can impact workers' MH. Positive psychology researchers, such as Seligman (2007), have also argued for the need for such workplace-related research. There is substantial scope for future research to consider how workers in MDI with existing anxiety and depression can be effectively supported within the workplace and for better quality studies on workplace MH promotion and prevention interventions (Barry et al., 2009). The European Agency for Safety and Health at Work has recently called for research on workers with mental disorders and the MH consequences of work demands and overload (European Agency for Safety and Health at Work, 2013). The present findings also support calls for systematic reviews with a health equity focus (Welch et al., 2012).

This study found a range of risk factors for anxiety and depression among workers in MDI, categorised as individual factors, team environment, work conditions and work–home interference. The predominant risk factors identified were work conditions and team environment, including job demands and poor workplace relationships. The findings support the need for workplace MH interventions and policies that are organisationally focused and that address structural factors. The findings also underscore the potential for primary prevention and early intervention strategies to improve the MH and well-being of a large proportion of the population while simultaneously increasing economic productivity (Dollard & Neser, 2013).
